# Supplementary material for: High Performance Gold Nanorods@DNA Self-Assembled Drug-Loading System for Cancer Thermo-Chemotherapy in the Second Near-Infrared Optical Window
Source: Pharmaceutics. 2022 May 23;14(5):1110. doi: 10.3390/pharmaceutics14051110 (PMC9145609; doi:10.3390/pharmaceutics14051110)
Supplement: Supplementary file 1 [file pharmaceutics-14-01110-s001.zip › pharmaceutics-1707641-supplementary.pdf]

Supplementary materials for:

High Performance gold nanorods@DNA Self-assembled Drug-loading System for Cancer Thermo-chemotherapy in the Second Near-Infrared Optical Window

Wei Chang[1,2#], Junfeng Wang[1#], Jing Zhang[1], Qing Ling[1,2], Yumei Li[3,*], and Jie Wang[1,*]

[1] Inflammation and Immune Mediated Diseases Laboratory of Anhui Province, School of Pharmacy, Anhui Medical University, Hefei 230032, China; changw@bbmc.edu.cn (W.C.); wjf2353258194@outlook.com (J.W.); zhangjing10087@outlook.com (J.Z.); lingqing22luan@163.com (Q.L.)

[2] Anhui Engineering Technology Research Center of Biochemical Pharmaceuticals, Faculty of Pharmacy, Bengbu Medical College, Bengbu 233030, China

[3] School of Basic Medicine, Gannan Medical University, Ganzhou 341000, China

* Correspondence: Yumei.li@gmu.edu.cn (Y.L.); 2020500067@ahmu.edu.cn (J.W.);

Tel.: +86-(13)-055271587 (Y.L.); +86-(13)-055165161176 (J.W.)

† These authors contributed equally to this work.

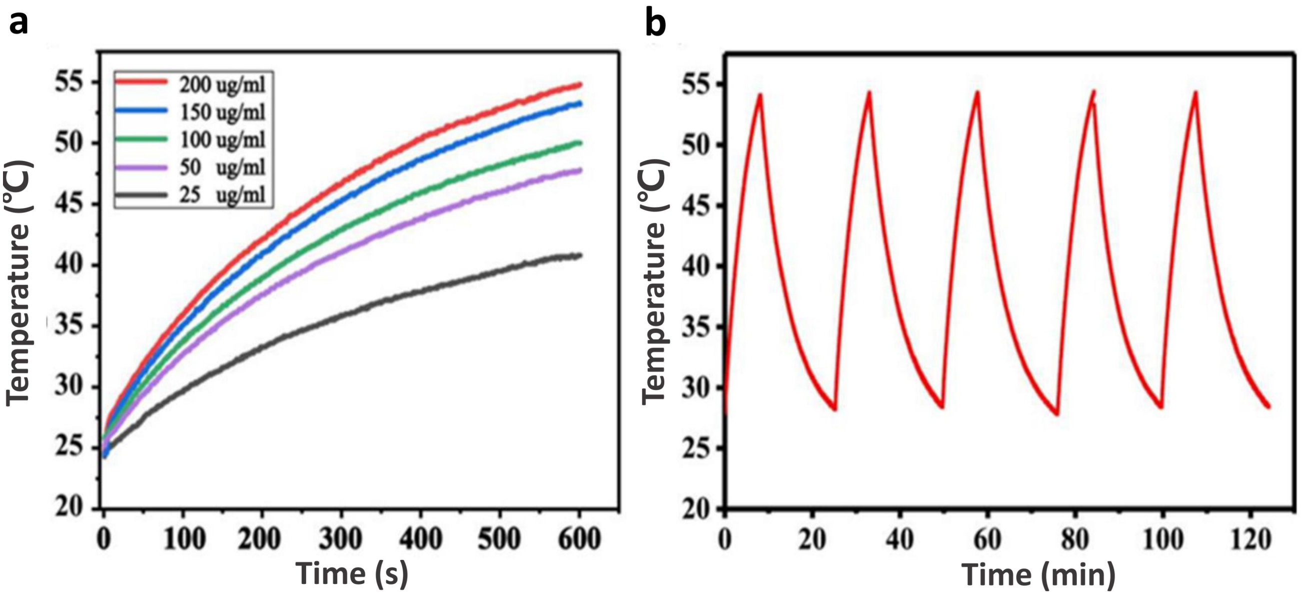

Figure S1. Photothermal efficiency analysis of GNR@CTA. (**a**) The photothermal performance of the GNR@CTA with different concentrations of Au irradiated for 10 min by 0.8 W·cm^{-2} 808 nm laser irradiation. (**b**) Heating and cooling circles for testing the stability of GNR@CTA (200 ug/ml) exposed to 0.8 W·cm^{-2} 808 nm laser irradiation.

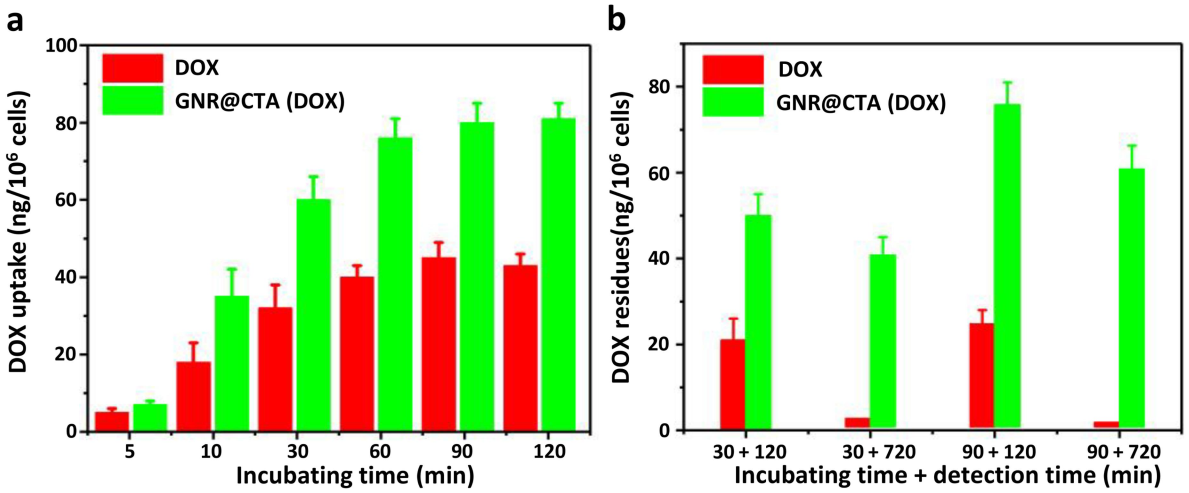

Figure S2. Photothermal efficiency analysis of GNP. (**a**) Compared the drug loading efficiency of DOX and GNR@CTA(DOX). (**b**) Compared the retention time of DOX in the cell.

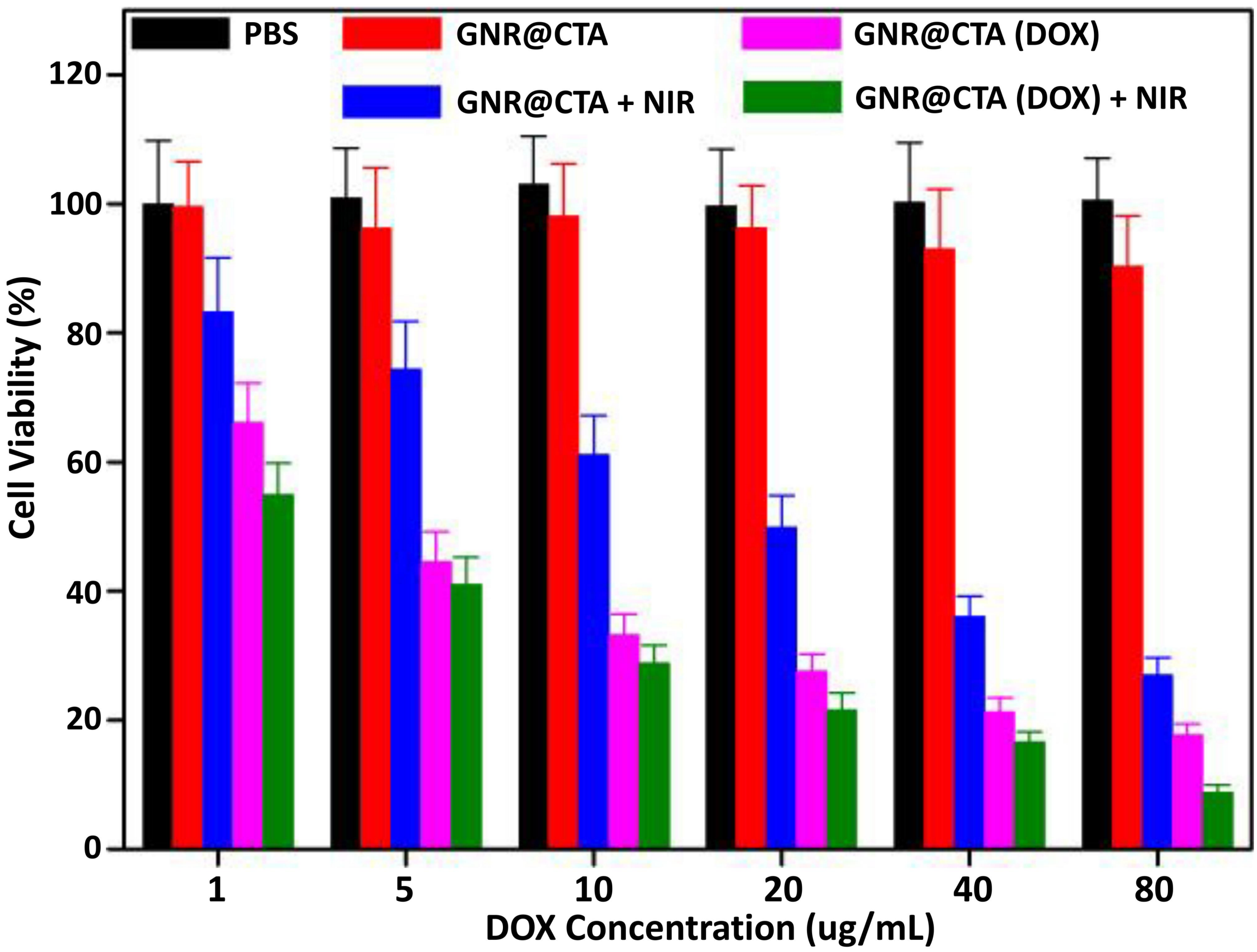

Figure S3. BT474 cells viability after incubation with different materials with or without 808 nm laser.

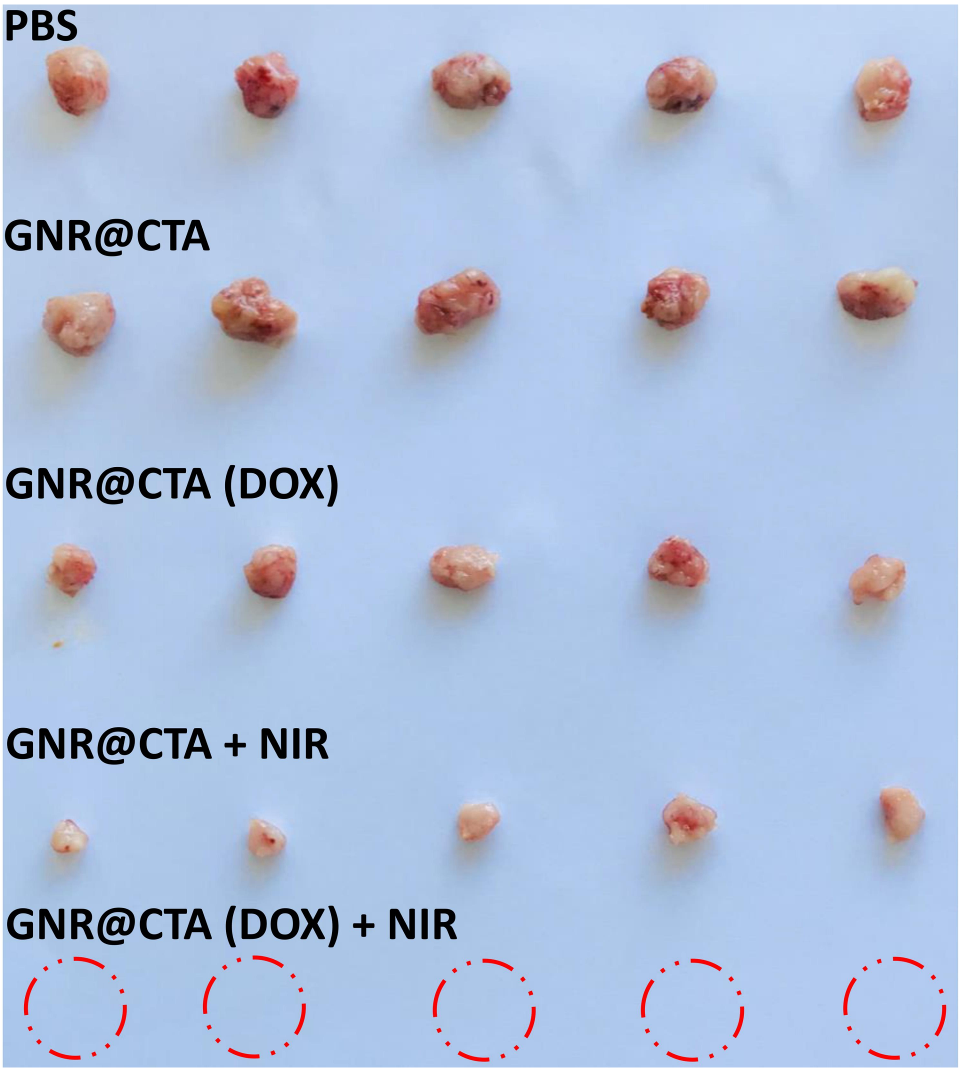

Figure S4. The photographs of dissected tumors from each group mice after different treatments.
